# Effective natural inhibitors targeting granzyme B in rheumatoid arthritis by computational study

**DOI:** 10.3389/fmed.2022.1052792

**Published:** 2022-12-13

**Authors:** Xinyu Wang, Ye Jiang, Pengcheng Zhou, Liangxin Lin, Yilin Yang, Qifan Yang, Jiting Zhang, Dong Zhu

**Affiliations:** Department of Orthopaedic Trauma, Center of Orthopaedics and Traumatology, The First Hospital of Jilin University, Changchun, China

**Keywords:** GZMB, RA, virtual screening, drug candidate, computer-aided technology

## Abstract

**Background:**

Rheumatoid arthritis (RA) is an autoimmune disease characterized by erosive arthritis, and current treatments for RA fall short of the outcomes expected by clinicians and patients.

**Objectives:**

This study aimed to identify novel therapeutic and prognostic targets in RA at the genomic level and to screen desirable compounds with potential inhibitory effects on GZMB.

**Methods:**

We performed differential gene analysis on GSE55235 and GSE55457 from Gene Expression Omnibus (GEO) and then obtained the intersection of the two differentially expressed genes (DEGs) lists by drawing Venn diagrams. Then we performed protein-protein interaction (PPI) network analysis, Gene Ontology (GO) analysis and Kyoto Encyclopedia of Genes and Genomes (KEGG) analysis on the DEGs of the intersection. Next, we downloaded the crystal structure of Granzyme B (GZMB). Molecular docking technology was used to screen potential inhibitors of GZMB in subsequent experiments, and we then analyzed the toxicity and water solubility of these potential inhibitors for future drug experiments. Finally, whether the docking of these small molecules with GZMB is stable is tested by molecular dynamics.

**Results:**

A total of 352 mutual DEGs were identified. Twenty hub genes were obtained according to PPI network analysis, among which the GZMB gene attracted the attention of our research. Three potent natural compounds, ZINC000004557101, ZINC000012495776, and ZINC000038143593, bound to GZMB, show better binding affinity. Furthermore, they are predicted to own low Ames mutagenicity, developmental toxicity potential, rodent carcinogenicity, and high tolerance to cytochrome P4502D6. Molecular dynamics simulations show that ZINC000004557101 and GZMB have more advantageous potential energy and can exist stably in a natural environment. Moreover, we finally verified the inhibitory effect of ZINC000004557101 on granzyme B by 3-(4,5-Dimethylthiazol-2-yl)-2,5-diphenyltetrazolium bromide (MTT) assay and Western blotting experiment.

**Conclusion:**

RA patients showed increased GZMB expression. ZINC000004557101 is a potential drug targeting GZMB for treating RA.

## Introduction

Rheumatoid arthritis (RA) is an inflammatory, chronic synovitis-based systemic disease of unknown etiology. It is featured by polyarticular, symmetrical, and aggressive joint inflammation of the facet joints of the feet and hands, often accompanied by extra-articular organ involvement and positive serum rheumatoid factor, which can lead to loss of function and joint deformity. RA affects 0.5–1.0% of adults worldwide, primarily in industrialized countries, with 5–50 per 100,000 persons annually. The disease is most representative in women and the elderly (1). At present, the primary treatment method for RA is glucocorticoid therapy. However, there are many side effects after long-term hormone therapy, such as osteoporosis, necrosis of the femoral head, obesity, full moon face, and low immunity, which lead to a severe decline in the quality of life of patients with RA (2, 3).

Bioinformatics and chip technology have been increasingly used to analyze molecular and genetic mechanisms in diseases with complicated clinical manifestations and poor prognoses (4). Bioinformatics analysis can identify essential driver genes and aberrant regulatory pathways of diseases and find their therapeutic targets accurately. Molecular docking and virtual screening are frequently used in the design of drugs (5, 6). They determine the binding capability of a protein to a ligand at the atomic level and calculate the pharmacological properties specific to various ligands (7, 8). Current treatments for RA do not meet the expectations of clinicians and patients. Therefore, we combined virtual screening analysis with bioinformatics to explore new therapies and screen effective drugs for RA (9).

Through bioinformatics analysis, we found that Granzyme B (GZMB) presented a state of high expression in RA. GZMB is a granzyme expressed by cytotoxic lymphocytes with the extracellular capacity to cleave extracellular matrix (ECM) components, cytokines, cellular receptors and coagulation proteins (10). GZMB is significantly elevated in chronic and inflammatory skin diseases, including diabetic ulcers, hypertrophic scarring, and skin aging, associated with its pro-apoptotic function (11). Clinical trials have shown that elevated serum GZMB levels are related to disease activity and joint damage in RA patients because GZMB induces chondrocytes apoptosis (12, 13).

This study aimed to combine virtual screening analysis with bioinformatics to screen novel and promising targeted agents for the treatment of RA. First, we used the GEO dataset to screen differentially expressed genes (DEGs) between RA and everyday individuals. Subsequently, we used these DEGs to establish a protein-protein interaction (PPI) network and screened the GZMB gene that plays a role in RA. Finally, we used a molecular docking approach to screen compounds that inhibit GZMB and assess their physicochemical properties. Subsequently, the inhibitory effect of ZINC000004557101 on Granzyme B was verified by MTT assay. Not only that, we treated fibroblasts in the six-well plate with different doses of ZINC000004557101, extracted total proteins by lysis of the cells, separated them by electrophoresis, transferred the proteins to the membrane, cleaved bands of interest, The concentration of Granzyme B decreased with the increase of ZINC000004557101 concentration. Candidate compounds and their pharmacological properties are listed, providing directions for developing GZMB inhibitors to treat RA.

## Results

### Identification of differentially expressed genes

**FIGURE 1:**
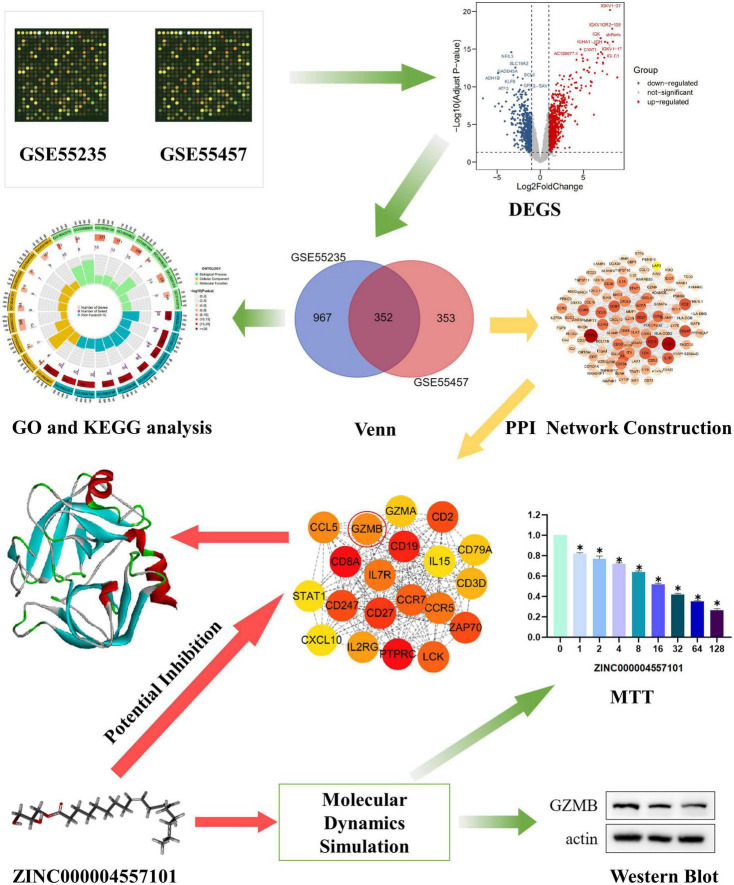
Research framework. The first set of images was chosen to represent the tissue dataset from the Gene Expression Comprehensive Database.

**FIGURE 2:**
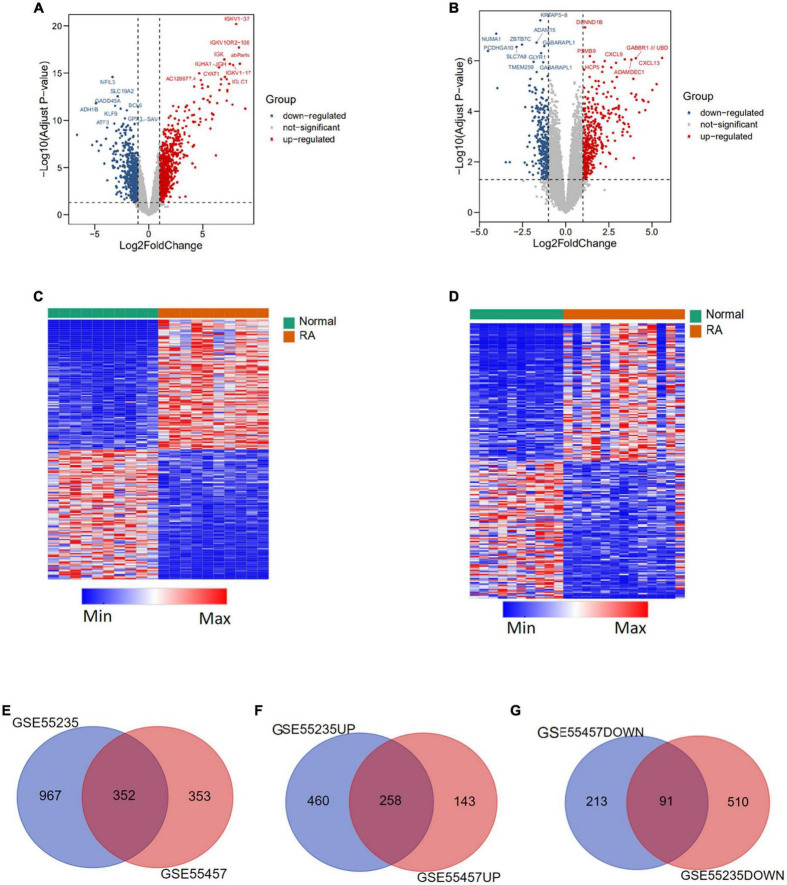
Differentially expressed genes (DEGs) between rheumatoid arthritis (RA) and normal tissues. **(A)** Volcano plot of differentially expressed genes in GSE55235 dataset. **(B)** Volcano plot of differentially expressed genes in GSE55457 dataset. **(C)** Heatmap of differentially expressed genes in GSE55235 dataset. **(D)** Heatmap of differentially expressed genes in GSE55457 dataset. **(E)** Veen plot of differentially expressed genes between GSE55235 and GSE55457. **(F)** Veen plot of up-regulated differential genes in two datasets. **(G)** Veen plot of downregulated differential genes in two datasets.

The whole process of data analysis is depicted in Figure 1. We screened DEGs from GSE55235 and GSE55457 datasets and plotted volcano plots and heatmaps (Figures 2A–D). A total of 1,319 DEGs were extracted from GSE55235, of which 718 were upregulated, and 601 were downregulated. Of the 705 DEGs found in GSE55457, 401 were upregulated, and 304 were downregulated. By performing Venn diagram analysis (Figures 2E–G), 352 mutual DEGs were identified in these two datasets, including 258 upregulated and 91 downregulated genes.

### Activation and route enrichment

**FIGURE 3:**
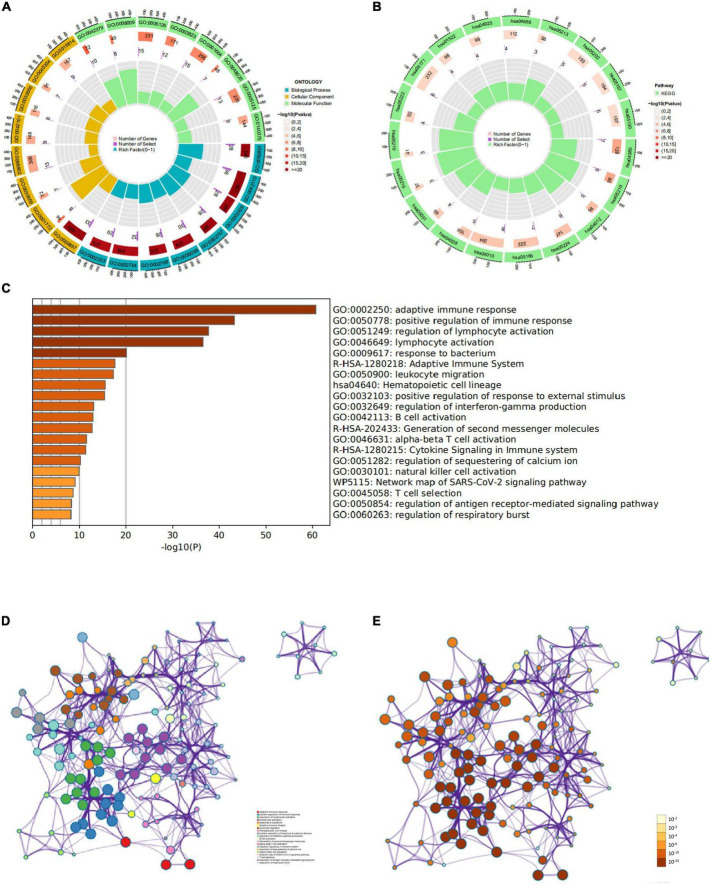
Functional and pathway enrichment analysis of upregulated genes. **(A)** Gene ontology (GO) enrichment analysis. **(B)** Kyoto encyclopedia of genes and genomes (KEGG) enrichment analysis. **(C)** Heatmap of Metascape enrichment analysis. **(D)** Differentially expressed genes (DEGs), colored by cluster ID. DEGs in the same cluster ID node are closely related to each other. **(E)** DEGs colored by *P*-value. Terms with more significant *P*-values contain more genes.

To understand the functions of these genes, we performed Metascape analysis for upregulated and downregulated genes common to both data sets. The upregulated genes were focused on “adaptive immune response,” “active immune response” and “adaptive immune system” (Figure 3). The downregulated genes enriched in the “piranha receptor pathway,” “response to growth factors and “vascular development. In addition, the upregulated genes are significantly enriched in “immune response-activating cell surface receptor signaling pathway,” “immunological synapse,” and “immune receptor activity” of gene ontology (GO) bioprocess analysis; in “Primary immunodeficiency,” “cytokine-cytokine receptor interactions,” and “viral protein-cytokine receptor interaction” of kyoto encyclopedia of genes and genomes (KEGG) pathways analysis (Supplementary Figure 1). Downregulated genes were significantly focused on “fat cell differentiation, “regulation of smooth muscle cell proliferation and “smooth muscle cell proliferation in GO biological process analysis; “Osteoclast differentiation,” “Colorectal cancer” and “Smooth muscle cell proliferation in KEGG pathway analysis. RA is a chronic inflammatory autoimmune disease (14), the immune system is closely related to the illness, and upregulated genes are inextricably linked to immunity; thus, upregulated differential genes play a vital role in such diseases.

### Construction of the protein-protein interaction network

**FIGURE 4:**
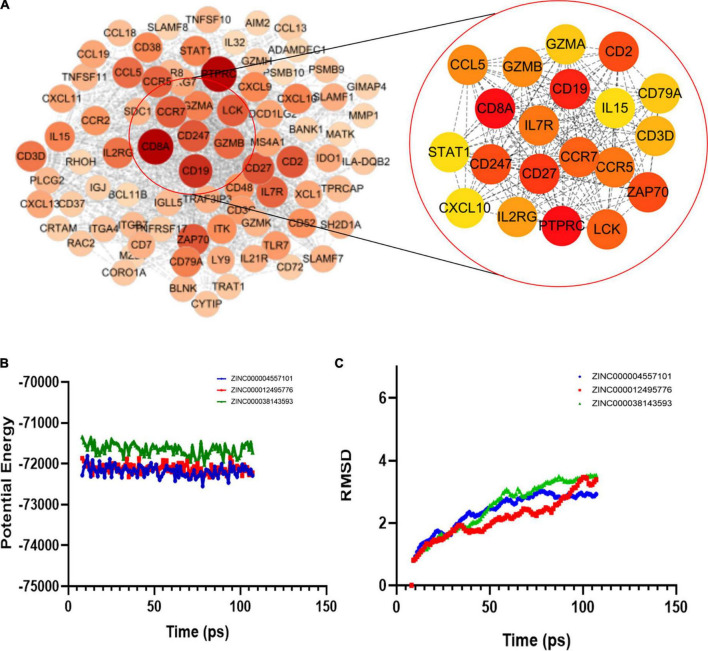
**(A)** Protein-protein interaction (PPI) network model of upregulated genes and top 20 gene associations. **(B)** Molecular dynamics simulation results of three complexes. Potential energy; **(C)** RMSD: root-mean-square deviation.

We then used the Search Tool for the Retrieval of Interacting Genes (STRING) to obtain the PPI network of upregulated genes (Figure 4A). Using Cytoscape software, we identified 20 hub genes with degree values: PTPRC, GZMB, CD19, CD27, CD2, ZAP70, CD247, CCR7, LCK, IL7R, CCR5, GZMB, CCL5, IL2RG, CD3D, CD8A, CD79A, IL15, STAT1, and CXCL10 (Table 1). GZMB has natural killer cell-like cytotoxicity in RA and can induce chondrocyte apoptosis (13). Clinical trials have shown that: RA disease activity and joint damage in patients are associated with elevated GZMB levels in serum (12). In addition, animal experiments have demonstrated that GZMB gene silencing can inhibit the mitogen-activated protein kinases (MAPK) signaling pathway by regulating the expression of inflammatory factors, apoptosis-related factors (BCL-2 and caspase) and angiogenesis-related factors [vascular endothelial-derived growth factor (VEGF) and basic fibroblast growth factor (bFGF)], thus alleviating synovial tissue hyperplasia and articular cartilage tissue damage caused by RA (15, 16). Therefore, GZMB was selected as the object of our study.

**TABLE 1:** Top 20 degrees genes among upregulated genes.

Gene	Degree	Betweenness	Gene	Degree	Betweenness
PTPRC	65	2745.82592	CCR5	42	428.87495
CD8A	65	1707.70276	GZMB	41	162.2748
CD19	54	1143.59629	CCL5	41	3989.54305
CD27	46	299.60786	IL2RG	39	470.6634
CD2	45	350.54633	CD3D	38	454.57177
ZAP70	45	606.61616	GZMA	36	198.9419
CD247	45	790.82431	CD79A	36	428.70041
CCR7	44	352.85474	IL15	35	224.33819
LCK	44	959.25884	STAT1	35	955.02005
IL7R	42	259.97414	CXCL10	35	454.5681

### Virtual small molecule inhibitors screening

As GZMB is a critical gene in the development of RA, we used virtual screening to discover the most effective small molecule inhibitors against GZMB to screen potential drugs for treating RA. Before this, we obtained the molecular structures of the GZMB protein and its inhibitors (Supplementary Figures 2A–C). Table 2 lists the top 20 compounds as determined by LibDock. ZINC000004557101, ZINC000012495776, and ZINC000038143593 have higher LibDock scores, showing their higher affinity to bind to GZMB.

**TABLE 2:** Top 20 ranked compounds with higher LibDock scores.

Name	Libdock score	Name	Libdock score
ZINC000014951634	175.897	ZINC000014951658	149.361
ZINC000044281738	167.457	ZINC000011616635	149.299
ZINC000005158610	155.28	ZINC000014946303	148.435
ZINC000011616633	153.839	ZINC000008214547	148.434
ZINC000085826837	153.733	ZINC000014767594	147.151
ZINC000014767590	153.395	ZINC000003938684	145.902
ZINC000012495776	152.793	ZINC000013374325	145.151
ZINC000044086691	152.001	ZINC000008234257	145.001
ZINC000038143593	151.849	ZINC000000689691	144.346
ZINC000004098643	150.066	ZINC000004557101	144.144

### Adsorption, distribution, metabolism, excretion, and toxicity characteristics evaluation

The adsorption, distribution, metabolism, excretion (ADME) module predicted the brain/blood barrier (BBB), human intestine absorption, water hepatotoxicity, plasma protein binding (PPB) characteristics, cytochrome P450 2D6 (CYP2D6) binding, and solubility (Table 3). Among these compounds, ZINC000004557101, ZINC000012495776, and ZINC000038143593 exhibit high levels of absorption and excellent solubility in water. High water solubility can promote the body’s exertion of efficacy and drug metabolism. At the same time, the safety of natural compounds is something we should also place a high priority on. We employed the TOP KAT module to research the toxicity of these 20 compounds, including their possible developmental toxicity, Ames mutagenicity, and hepatotoxicity testing (Table 4). Among the results, it was found that 12 compounds were non-mutagenic, and five combinations had no developmental toxicity. Compared to the remaining compounds, ZINC000004557101, ZINC000012495776, and ZINC000038143593 had low levels of rodent carcinogenicity, hepatotoxicity, AMES mutagenicity, and developmental toxicity. As a result, they are considered non-toxic and safe medications that deserve future investigation. Not only that, but we also performed a drug group analysis for these three compounds (Supplementary Figures 2D–F).

**TABLE 3:** Adsorption, distribution, metabolism, and excretion properties of compounds.

Compounds	Solubility level	BBB level	CYP2D6	Hepatotoxicity	Absorption level	PPB level
ZINC000014951634	3	4	0	0	3	0
ZINC000044281738	0	4	0	1	3	1
ZINC000005158610	2	4	1	0	2	1
ZINC000011616633	2	4	0	0	3	0
ZINC000085826837	2	4	0	0	2	0
ZINC000014767590	0	4	0	0	3	1
ZINC000012495776	4	3	0	0	0	1
ZINC000044086691	1	4	0	0	3	1
ZINC000038143593	3	4	0	0	3	0
ZINC000004098643	2	4	0	0	2	1
ZINC000014951658	3	4	0	0	3	0
ZINC000011616635	2	4	0	0	3	0
ZINC000014946303	0	4	0	1	3	0
ZINC000008214547	2	4	0	0	0	0
ZINC000014767594	0	4	0	0	3	1
ZINC000003938684	3	4	0	1	3	1
ZINC000013374325	2	2	0	0	0	0
ZINC000008234257	3	4	0	0	2	0
ZINC000000689691	2	4	1	1	2	1
ZINC000004557101	3	4	0	0	1	0

**TABLE 4:** Toxicities of compounds.

Compounds	Mouse NTP		Rat NTP		Ame’s	DTP
	Female	Male	Female	Male		
ZINC000014951634	0.089	0	1	0	0	1
ZINC000044281738	0	0	1	0	0.007	0
ZINC000005158610	0	1	1	0	1	1
ZINC000011616633	0	1	1	1	1	1
ZINC000085826837	0.186	1	1	0.998	0	1
ZINC000014767590	0.494	0	0	1	0	0
ZINC000012495776	0.891	0.056	0	0	0.009	0
ZINC000044086691	1	0	0.987	0.998	0.004	1
ZINC000038143593	0.061	0	0.274	0.088	0	1
ZINC000004098643	0.997	0	1	0	0	0.995
ZINC000014951658	1	0	1	0	0	1
ZINC000011616635	0	1	1	1	1	1
ZINC000014946303	0.991	1	0	1	0	0.007
ZINC000008214547	1	0	0.983	0	0.001	1
ZINC000014767594	0.494	0	0	1	0	0
ZINC000003938684	0.025	0.953	1	0.026	0	1
ZINC000013374325	0.002	0	1	0.015	0	0.095
ZINC000008234257	1	0.705	0	0.373	0	1
ZINC000000689691	0	1	1	0.008	1	1
ZINC000004557101	0	0.006	0	0	0	0

### Analyses of ligand binding sites and molecular dynamics simulations

**FIGURE 5:**
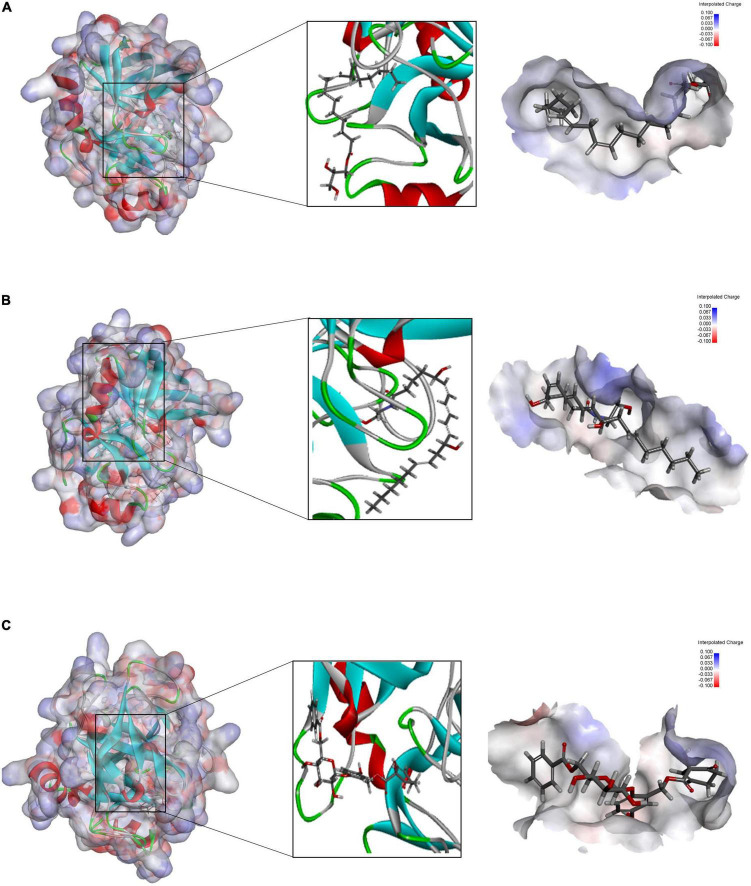
**(A)** Schematic representation of the interaction between ZINC000004557101 and granzyme B (GZMB). **(B)** Schematic representation of the interaction between ZINC000012495776 and GZMB. **(C)** Schematic representation of the interaction between ZINC000038143593 and GZMB.

In the COCKER module, we attach ZINC000004557101, ZINC000038143593, and ZINC000012495776 one by one to the molecular structure of GZMB (Figure 5) and start to calculate the COCKER potential (Table 5). The CDOCKER potential of ZINC000012495776 is lower than ZINC000038143593 and ZINC000004557101, and GZMB can bind to ZINC000004557101, ZINC000038143593, and ZINC000012495776 with high affinity. Hydrogen bonding and π-related interactions can be calculated and found in the structures of compounds ZINC000004557101, ZINC000038143593, and ZINC000012495776, which all show several bond acceptor and donor atoms (Tables 6, 7). In the complexes, they form several pairs of π-related interactions and hydrogen bonds with GZMB (Supplementary Figure 3). The RMSD curves and potential energies of various small-molecule compounds, GZMB complexes showed that the complex trajectory of ZINC000004557101 with GZMB reached equilibrium after 85 ps, and the complex possible points stabilized over time (Figures 4B,C). The remaining two compounds did not reach equilibrium.

**TABLE 5:** CDOCKER potential energy of compounds with granzyme B.

Compounds	-CDOCKER potential energy (kcal/mol)
ZINC000004557101	52.5355
ZINC000012495776	48.9419
ZINC000038143593	60.6626

**TABLE 6:** Hydrogen bond interaction parameters for each compound with granzyme B residues.

Receptor	Compound	Donor atom	Receptor atom	Distances (Å)
GZMB	ZINC000012495776	LYS40:HZ2	ZINC000012495776:O42	1.93637
		GLY193:HN	ZINC000012495776:O54	2.15137
		SER195:HN	ZINC000012495776:O54	2.76539
		SER195:HG	ZINC000012495776:O54	1.82105
		ARG226:HH21	ZINC000012495776:O63	2.15531
	ZINC000004557101	ARG217:HH12	ZINC000004557101:O62	1.82764
		ARG217:HH22	ZINC000004557101:O62	2.22586
	ZINC000038143593	HIS57:HD1	UNK0:O3	2.53121
		SER214:HG	UNK0:O13	2.23965
		GLY216:HN	UNK0:O19	2.11862
		ARG226:HH22	UNK0:O27	2.43503
		UNK0:H46	ASP102:OD2	1.77564

**TABLE 7:** π-related interaction parameters for each compound with granzyme B.

Receptor	Compound	Donor atom	Receptor atom	Distances (Å)
GZMB	ZINC000012495776	ZINC000012495776:C4	LEU172	4.39543
		TYR174	ZINC000012495776:C4	4.70693
	ZINC000004557101	ZINC000004557101:C4	VAL213	4.58891
	ZINC000038143593	VAL213	ZINC000038143593	4.87385

### ZINC000004557101 reduces proliferation of rheumatoid arthritis synovial fibroblasts and granzyme B expression

**FIGURE 6:**
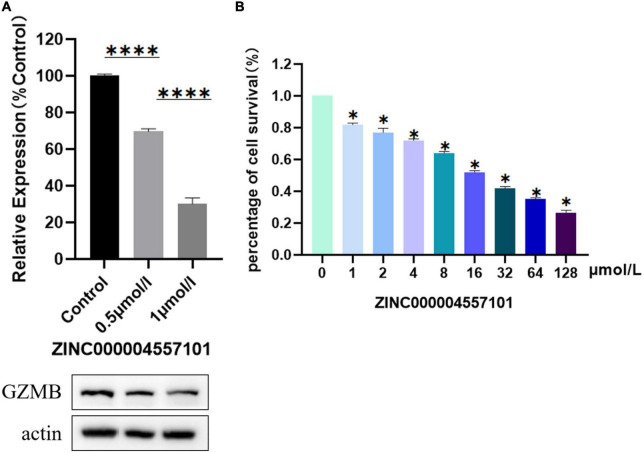
Results of 3-(4,5-Dimethylthiazol-2-yl)-2,5-diphenyltetrazolium bromide (MTT) assay, and Western blotting of compounds ZINC000004557101. **(A)** Western blotting. **(B)** MTT assay.

We investigated the effect of ZINC000004557101 on synovial fibroblasts from RA rats. MTT assay showed that the higher the drug dose, the lower the activity of fibroblasts (*P* < 0.05) (Figure 6B).

To verify whether ZINC000004557101 could inhibit GZMB in fibroblasts, we assessed GZMB levels using Western blotting. Western blotting showed that the expression of GZMB decreased with increasing drug concentration (Figure 6A).

## Discussion

At present, RA is one of the most serious teratogenic diseases in the world. Once it occurs, the severe pain will affect the patient’s everyday life. Patients with RA may need to rely on analgesics for a long time, resulting in damage to multiple organs throughout the body, including the digestive tract, lungs, heart, and nervous system (17). Tumor necrosis factor (TNF) inhibitors are drugs selected by high-throughput screening to treat RA (18). Although these small molecules have better safety and lower production costs than existing drugs, long-term clinical trials have shown that: TNF inhibitors are associated with an increased risk of infection, especially early in the course of treatment, which puts patients with RA at a higher risk of disease (including tuberculosis) after using these drugs (19). Therefore, we need to investigate new safe and effective drugs to treat RA.

Bioinformatics and structural biology tools have been employed efficiently and systematically in recent years to find particular targets for complicated clinical disorders (20). These tools were used in this research to target inhibition of genes that play an important role in the disease, so the findings have precision for treating the disease. Bioinformatics analysis of GSE55235 and GSE55457 gene expression patterns was performed in this study. Following the intersection of upregulated genes using a Venn diagram, a PPI network for the intersecting upregulated genes was built. Eventually we chose GZMB as a therapeutic target for RA.

Granzyme B is a serine protease in the cytoplasm of cytotoxic T lymphocytes and natural killer cells. It is involved in the induction of apoptosis in target cells. (15, 16) And It has been associated with a high incidence of autoimmune diseases, rectal cancer, breast cancer and other cancers (21–24). It has been shown that inhibition of MAPK signal transduction by GZMB silencing can reduce the expression of inflammatory factors, apoptosis-related factors (Bcl-2 and caspase) and angiogenesis-related factors (VEGF and bFGF), thus reducing the synovial tissue hyperplasia and articular cartilage tissue damage associated with RA (16). Based on the results of bioinformatics analysis, we selected GZMB as our research gene. (21). Animal experiments showed that GZMB gene silting could alleviate synovial tissue hyperplasia and articular cartilage tissue damage caused by RA (25). Clinical trials have shown elevated levels of GZMB in the synovial and plasma fluid of patients with RA. (26, 27) Although the current GZMB small molecule inhibitor VTI-1002 shows high specificity, potency, and target selectivity, it is only a novel approach for the treatment of skin burn wounds and is not an effective treatment for patients with rheumatoid arthritis (28).

According to the results of LibDock, a total of 9,467 molecules had a strong binding affinity for GZMB. The tops 20 small compounds were elected for further investigation. Following that, we look at the pharmacological and toxicological aspects of small compounds in more detail. The results revealed that ZINC000004557101, ZINC000012495776, and ZINC000038143593 were the most potential GZMB inhibitors. In the first place, their levels of water solubility and intestinal absorption are optimal. Furthermore, these compounds do not appear to interfere with the action of CYP2D6 (cytochrome P450 2D6). They are non-toxic to the liver. They also show minimal developmental toxicity, Ame’s mutagenicity, and rodent carcinogenicity, among other characteristics. As a result, these compounds are effective inhibitors of the GZMB. ZINC000004557101, ZINC000012495776, and ZINC000038143593 are potential GZMB inhibitors that are safe to use. As a result, we went one step further. Aside from that, we looked into the binding of these compounds to the GZMB.

The CDOCKER module can well reflect candidate compounds’ bonding mechanisms and chemical bonds. As shown in Table 4, ZINC000004557101 and ZINC000012495776 have lower CDOCKER potential energy than ZINC000038143593, which may indicate that these two compounds bind GZMB with better affinity.

The final findings indicated that these small compounds we chose bind to GZMB protein rapidly and stably. As a result, they were selected for the following phase study. Following that, we ran molecular dynamics simulations. The RMSD and potential energy curves of the complex of Zn000004557101 and GZMB gradually stabilized and reached an equilibrium close to 100 Ps. However, the RMSD curves of the other two compounds and GZMB failed to reach equilibrium, so we predicted that ZINC000004557101 would be a better potential drug for RA than these two small molecule compounds.

We verified the effectiveness of the drug ZINC000004557101 through cytological experiments. In the MTT experiment, we set up nine groups of drug concentration gradients. Increasing drug concentrations inhibited the proliferation and activity of fibroblasts from RA rats synovium. In the Western blotting experiment, we proved that ZINC000004557101 could inhibit the expression of GZMB.

Finally, our research aimed to identify a potential GZMB inhibitory treatment candidate. This study was painstakingly designed and carried out with exact measurements, but we must admit that it had significant shortcomings. No medicine can be sold without being developed and improved. Several groups and atoms that can affect the pharmacological characteristics of the pharmaceuticals must be changed to improve this compound’s suitability as a therapeutic candidate. To confirm our findings, we will further investigate the specificity and sensitivity of ZINC000004557101 against GZMB in RA cell lines and RA animal models, as well as other pharmacological safety indicators such as MTD (Maximum Tolerated Dosage) and AB (Aerobic Biodegradability). Our future research will focus on these restrictions.

## Conclusion

Differential gene analysis was performed on data sets GSE55235 and GSE55457, and 258 common upregulated genes were obtained by Venn diagram. After the construction of the PPI network, GZMB is considered a critical therapeutic target for RA. ZINC000004557101 was screened as a drug candidate by computer-aided chemical and structural techniques (toxicity index, ADME, virtual screening, molecular docking, and kinetic simulation) from many natural drugs that may inhibit GZMB function. It has a high affinity for GZMB, competitively inhibits GZMB and reduces GZMB expression in fibroblasts from RA rats synovium. It also inhibited fibroblast proliferation in RA rats synovium, and the inhibition was positively correlated with the drug concentration. Therefore, ZINC000004557101 may become a promising, safe and reliable drug for RA treatment shortly with the deepening of experiments.

## Materials and methods

### Genetic data source

GSE55235 and GSE55457 gene expression data whose detection platforms were identical (GPL96, HG-U133A) were obtained from the Gene Expression Omnibus^1^. There are 20 regular and 23 patients with RA (29). Background correction and standardized analysis were performed on the microarray data.

### Identification of differentially expressed genes

The limma package was used to identify DEGs based on comparing expression values between standard and RA samples (20). The following were the screening criteria for DEGs: log2 fold change (FC) greater than 1 or less than -1, and the adjusted *p*-value is 0.05. The analysis result was presented by heatmap and volcano map drawn in RStudio software (version:4.1.2). A Venn diagram analysis of DEGs was performed between upregulated, downregulated and total DEGs^2^.

### Gene ontology and kyoto encyclopedia of genes and genomes pathway enrichment analyses of differentially expressed genes

Metascape is a website dedicated to visualizing, annotating, and characterizing genes. We uploaded the upregulated DEGs to this website and examined their enrichment in GO terms and signaling pathways. In addition, using the “clusterProfiler” R package, analyses of the GO and KEGG were carried out to enrich related trails.

### Protein-protein interaction network construction and module selection

STRING^3^ was utilized for bioinformatics PPI network analysis. Afterward, using Cytoscape software, hub genes and modules were identified utilizing complex molecular identification. This program is used to identify hub genes and their extent.

### Crystal structure of granzyme B

The ligand-binding pocket region of GZMB was chosen as the binding location for possible inhibitors of this enzyme. Virtual filtering was accomplished using Discovery Studio 4.5’s LibDock module (30). Discovery Studio 4.5 is a comprehensive molecular modeling and environmental simulation software launched by bio *via*. This software is commonly used in small molecule drug screening, allowing researchers to explore the best drug candidates in the vast drug market. This is because it has many functions, such as simulation/analysis, 3D molecular construction and 3D mapping. GZMB’s crystal structure was obtained from the PDB database. By eliminating crystalline water and other heteroatoms, protein structures were produced. Energy minimization was accomplished using the CHARMM force field and the Smart Minimizer method (31).

### Adsorption, distribution, metabolism, excretion, and toxicity prediction

Discovery Studio 4.5’s ADME module was used to determine the metabolism, excretion, distribution, and absorption of chosen substances. The TOP KAT module of Discovery Studio 4.5 was used to determine the selected compounds’ developmental toxicity, blood-brain barrier permeability, water solubility, liver toxicity, CYP2D6 inhibition, rodent carcinogenicity, human intestinal absorption, plasma protein binding, and Ame’s mutagenicity.

### Molecular docking

The CDOCKER module of Discovery Studio 4.5 was employed for the molecular docking study. COCKER is capable of producing exact molecular docking findings using the CHARMM field. The ligand is permitted to bend while the receptor stays stiff during docking. The CHARMM energy (interaction energy plus ligand strain) and interaction energy are utilized to determine the ligand-binding affinity for each complex posture. Generally, crystallized water molecules are eliminated among rigid and semi-flexible docking processes since they may interfere with the formation of receptor-ligand complexes (32, 33). The addition of hydrogen atoms then modifies the proteins. Based on each test molecule’s COCKER interaction energy, different postures may be studied. To illustrate the combination pattern’s dependability, the potent tetrapeptide aldehyde inhibitor a human granzyme B with non-covalently bound inhibitor (AcIEPD-CHO) was removed from the binding site and then realigned into the crystalline structure of GZMB.

### Molecular dynamics simulation

We selected small molecule and granular enzyme B with better structure during this process. First, we constructed an orthogonal box with a small compound and a particulate B complex. It is then solved using a model of solvated water with distinct periodic boundaries. Additionally, solid chloride with an ionic strength of 0.145 was introduced into the physiological environment. Additionally, we assign a CHARMm force field to the system. We then reduced its energy consumption (the steepest down to 500 steps, a conjugate gradient of 500 degrees). The system’s temperature was increased from 50 to 300 K in the equilibrium simulations of 200 and 250 s. The time step is set to two frames per second. This process is performed using atmospheric pressure and temperature (NPT). The temperature was kept constant at 300 K. In addition, the remote static power was evaluated using the PME (particle grid Ewald) method, and the library of integrated network-based cellular signatures (LINCS) algorithm was modified to correct all the critical points related to hydrogen (34).

### Cell lines

Synovial fibroblasts from mice with RA were cultured in Dulbecco’s modified Eagle (DMEM, Hyclone, New York, NY, USA) supplemented with ten per cent fetal bovine serum. At 37^°^C, the petri dish contains 95% air and 5% carbon dioxide.

### 3-(4,5-Dimethylthiazol-2-yl)-2,5-diphenyltetrazolium bromide assay

Fibroblasts were seeded into 96-well dishes at a density of 500 cells/well and treated with different doses of ZINC000004557101. Cell viability was detected by dissolving MTT (Sigma, St. Louis, MO, USA) in 5 mg/ml phosphate buffer. On the day of the experiment, it was replaced with fresh DMEM supplemented with 10% fetal bovine serum and diluted MTT (1:10, 10% MTT) and incubated at 37^°^C for 3.5 h. Next, the dimethyl sulfoxide crystals were dissolved in a solution of 200 L of diethyl sulfoxide, and the medium was removed. The drop in MTT was measured using an ELx800 absorber enzyme (VT, USA) with absorbance at 570 nm.

### Western blotting

Fibroblasts in six-well plates were treated with different doses of ZINC000004557101. Prior to this, we selected cells in the logarithmic growth phase and seeded them into six-well plates at a density of 2 × 10 5 cells/well. After 48 h of culture, we extracted total protein by lysing cells and separated them by electrophoresis. The protein was transferred to the membrane and the band of interest was cleaved, treated with primary antibodies against GZMB and beta actin, then the membrane was washed and incubated with secondary antibodies. *Via* an enhanced chemiluminescence detection system (Pierce; Thermo Fisher Scientific).

### Statistics

All data were entered into SPSS 18.0 (SPSS Inc.,Chicago, IL, USA) for statistical analysis. Independent-samples *t*-tests were conducted, and *P*-values <0.05 were considered significant.

## Data availability statement

The datasets presented in this study can be found in online repositories. The names of the repository/repositories and accession number(s) can be found in the article/Supplementary material.

## Author contributions

DZ and JZ designed the experiments. YJ wrote the manuscript. QY, LL, and YY carried out experiments. PZ analyzed the results of the experiments. XW supervised the study and contributed to the data analysis. All authors contributed to the article and approved the submitted version.

## Footnotes

1
http://www.ncbi.nlm.nih.gov/geo

2
http://bioinformatics.psb.ugent.be/webtools/Venn/

3
http://string.embl.de/

## Supplementary material

Supplementary Figure 1 Functional and pathway enrichment analysis of downregulated genes. **(A)** Gene ontology (GO) enrichment analysis. **(B)** Kyoto encyclopedia of genes and genomes (KEGG) enrichment analysis. **(C)** Heatmap of Metascape enrichment analysis. **(D)** Differentially expressed genes (DEGs), colored by cluster ID. DEGs in the same cluster ID node are closely related to each other. **(E)** DEGs colored by *P*-value. Terms with more significant *P*-values contain more genes.

Supplementary Figure 2 **(A)** Molecular structure of GZMB. **(B)** Complex structure of granzyme B (GZMB) and a human granzyme B with non-covalently bound inhibitor (AcIEPD-CHO). **(C)** The initial complex structure is shown, with complex surfaces added. Blue represents positive charge and red represents negative charge. **(D–F)** Pharmacophore prediction using three-dimensional quantitative structure-activity relationship (3D-QSAR). **(D)** ZINC000004557101: Green represents hydrogen acceptor, blue represents hydrophobic center, and purple represents hydrogen donor. **(E)** ZINC000012495776: Green represents hydrogen acceptor, blue represents hydrophobic center, and purple represents hydrogen donor. **(F)** ZINC000038143593: Green represents hydrogen acceptor, blue represents hydrophobic center, purple represents hydrogen donor, and gold represents benzene ring.

Supplementary Figure 3 **(A)** The inter-molecular interaction of the predicted binding modes of ZINC000004557101 to granzyme B (GZMB); **(B)** ZINC000012495776 to GZMB, **(C)** ZINC000038143593 to GZMB.
