# Supplementary figures and images for: Effective natural inhibitors targeting granzyme B in rheumatoid arthritis by computational study
Source: Front Med (Lausanne). 2022 Dec 13;9:1052792. doi: 10.3389/fmed.2022.1052792 (PMC9792495; doi:10.3389/fmed.2022.1052792)

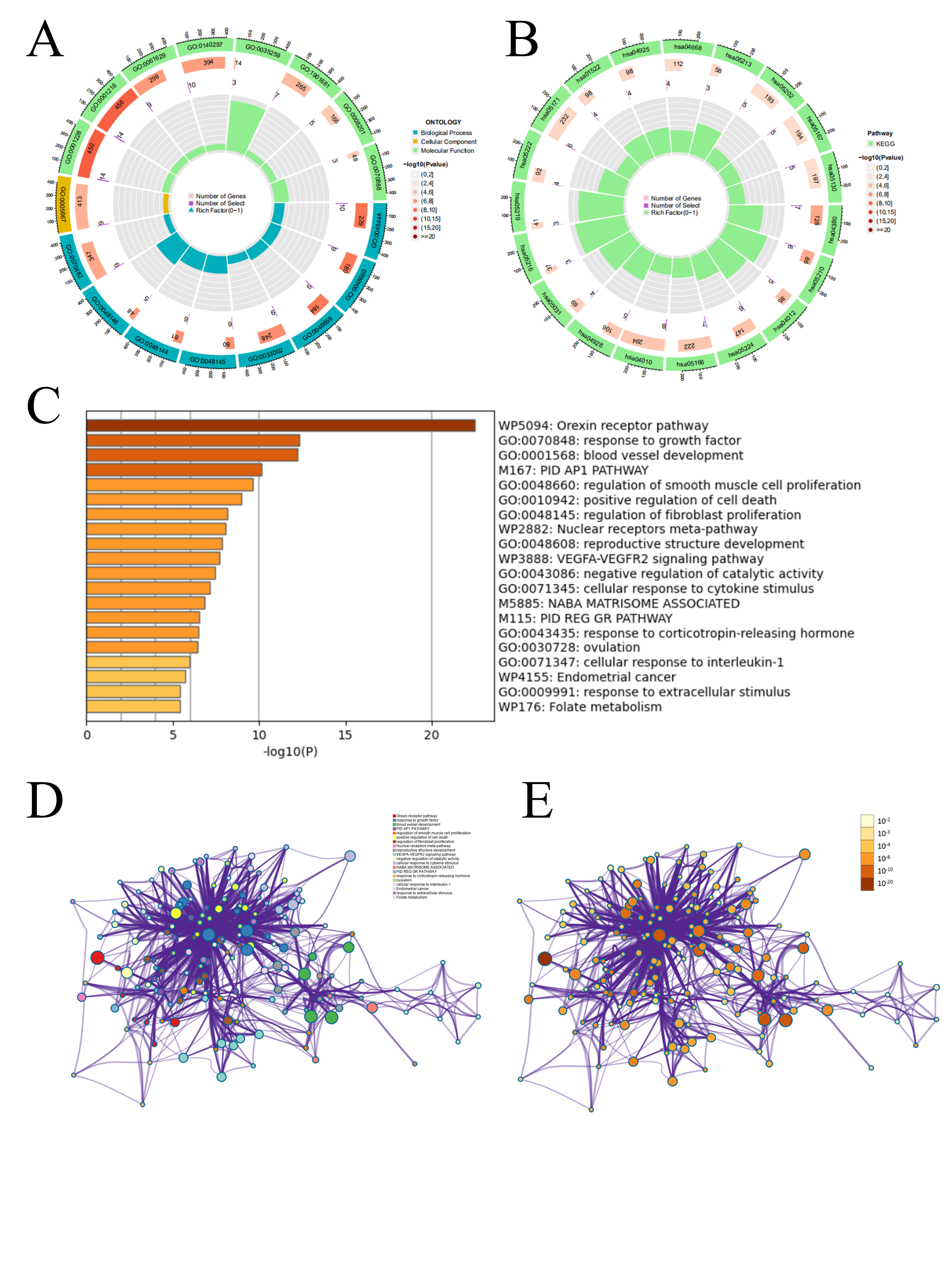

Supplement: Supplementary Figure 1 — Functional and pathway enrichment analysis of downregulated genes. (A) Gene ontology (GO) enrichment analysis. (B) Kyoto encyclopedia of genes and genomes (KEGG) enrichment analysis. (C) Heatmap of Metascape enrichment analysis. (D) Differentially expressed genes (DEGs), colored by cluster ID. DEGs in the same cluster ID node are closely related to each other. (E) DEGs colored by P-value. Terms with more significant P-values contain more genes. [file Image_1.TIF]

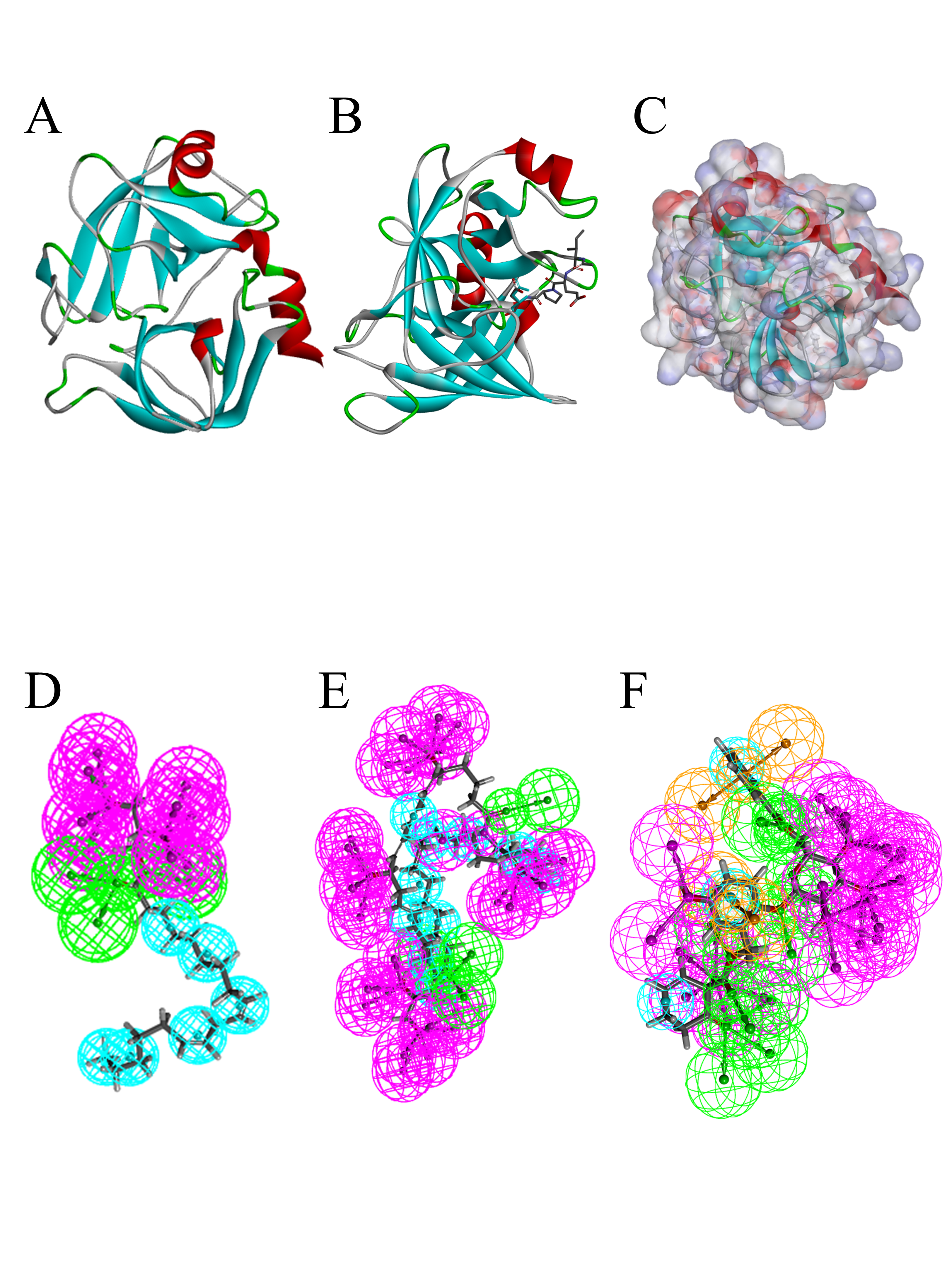

Supplement: Supplementary Figure 2 — (A) Molecular structure of GZMB. (B) Complex structure of granzyme B (GZMB) and a human granzyme B with non-covalently bound inhibitor (AcIEPD-CHO). (C) The initial complex structure is shown, with complex surfaces added. Blue represents positive charge and red represents negative charge. (D–F) Pharmacophore prediction using three-dimensional quantitative structure-activity relationship (3D-QSAR). (D) ZINC000004557101: Green represents hydrogen acceptor, blue represents hydrophobic center, and purple represents hydrogen donor. (E) ZINC000012495776: Green represents hydrogen acceptor, blue represents hydrophobic center, and purple represents hydrogen donor. (F) ZINC000038143593: Green represents hydrogen acceptor, blue represents hydrophobic center, purple represents hydrogen donor, and gold represents benzene ring. [file Image_2.TIF]

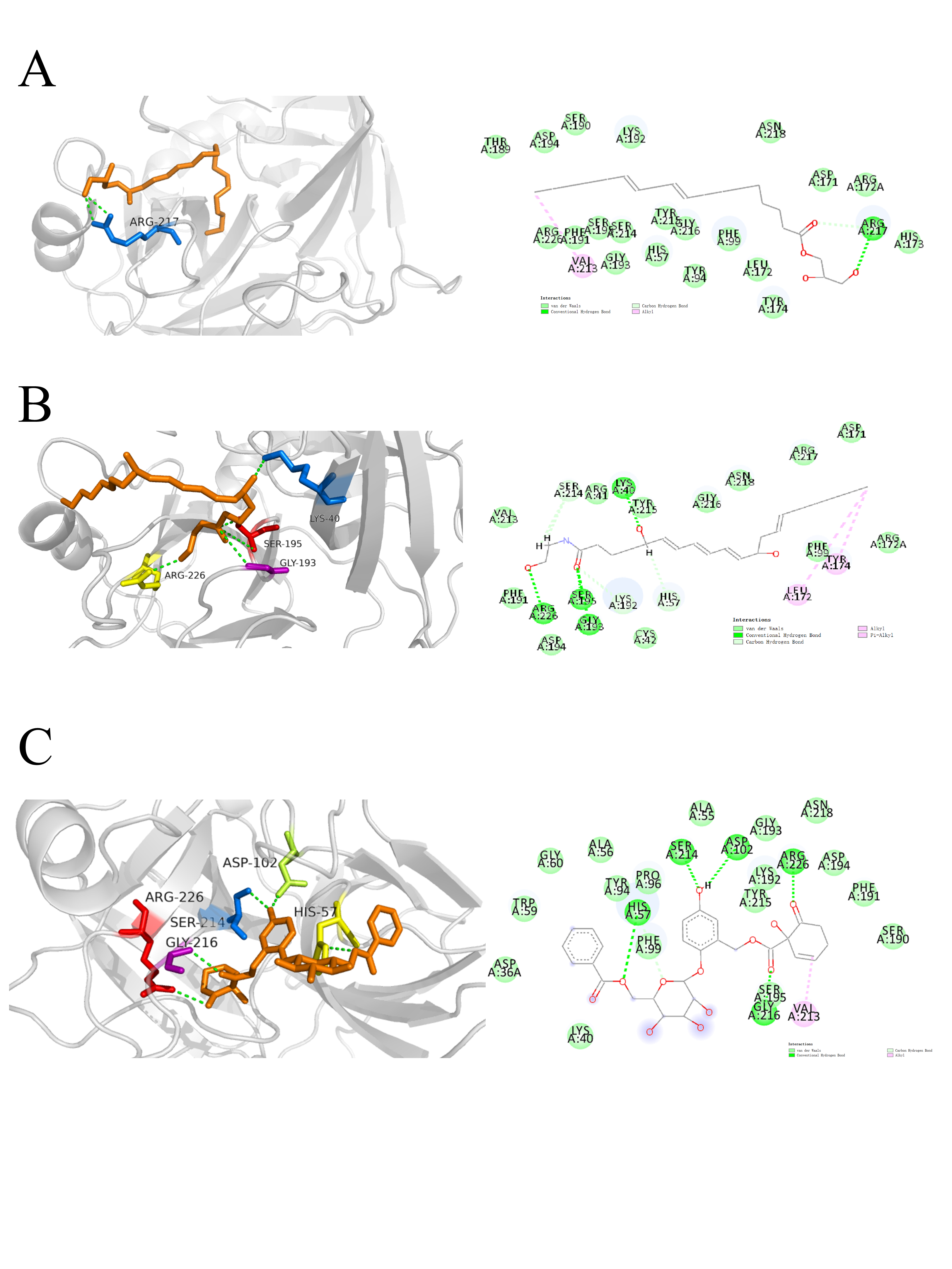

Supplement: Supplementary Figure 3 — (A) The inter-molecular interaction of the predicted binding modes of ZINC000004557101 to granzyme B (GZMB); (B) ZINC000012495776 to GZMB, (C) ZINC000038143593 to GZMB. [file Image_3.TIF]
